# National Institute of Allergy and Infectious Disease (NIAID) Funding for Studies of Hospital-Associated Bacterial Pathogens: Are Funds Proportionate to Burden of Disease?

**DOI:** 10.1186/2047-2994-1-5

**Published:** 2012-01-26

**Authors:** Seunghyug Kwon, Marin L Schweizer, Eli N Perencevich

**Affiliations:** 1 Iowa City Veterans Affairs Health Care System, Iowa City, IA USA; 2 Department of Epidemiology, College of Public Health, University of Iowa, Iowa City, IA USA; 3 Department of Internal Medicine, Carver College of Medicine, University of Iowa, Iowa City, IA USA

**Keywords:** Antibiotic resistance, NIH, Hospital-associated infection, research funding, disease burden

## Abstract

**Background:**

Hospital-associated infections (HAIs) are associated with a considerable burden of disease and direct costs greater than $17 billion. The pathogens that cause the majority of serious HAIs are *Enterococcus faecium, Staphylococcus aureus, Clostridium difficile, Klebsiella pneumoniae, Acinetobacter baumannii, Pseudomonas aeruginosa*, and Enterobacter species, referred as ESCKAPE. We aimed to determine the amount of funding the National Institute of Health (NIH) National Institute of Allergy and Infectious Diseases (NIAID) allocates to research on antimicrobial resistant pathogens, particularly ESCKAPE pathogens.

**Methods:**

The NIH Research Portfolio Online Reporting Tools (RePORT) database was used to identify NIAID antimicrobial resistance research grants funded in 2007-2009 using the terms "antibiotic resistance," "antimicrobial resistance," and "hospital-associated infection."

**Results:**

Funding for antimicrobial resistance grants has increased from 2007-2009. Antimicrobial resistance funding for bacterial pathogens has seen a smaller increase than non-bacterial pathogens. The total funding for all ESKCAPE pathogens was $ 22,005,943 in 2007, $ 30,810,153 in 2008 and $ 49,801,227 in 2009. *S. aureus *grants received $ 29,193,264 in FY2009, the highest funding amount of all the ESCKAPE pathogens. Based on 2009 funding data, approximately $1,565 of research money was spent per *S. aureus *related death and $750 of was spent per *C. difficile *related death.

**Conclusions:**

Although the funding for ESCKAPE pathogens has increased from 2007 to 2009, funding levels for antimicrobial resistant bacteria-related grants is still lower than funding for antimicrobial resistant non-bacterial pathogens. Efforts may be needed to improve research funding for resistant-bacterial pathogens, particularly as their clinical burden increases.

## Background

The National Institutes of Health (NIH) has been successful over the past 60 years in funding research that has greatly advanced modern medicine. However, given limited resources, many have begun to question how these research funds are allocated to various diseases.[1-3] The NIH has listed five major criteria for the allocation of research funds: public health needs, the scientific quality of research, the probability of success, the maintenance of a diverse portfolio, and the maintenance of an adequate scientific infrastructure.[4] While the Institute of Medicine panel has embraced these criteria as an appropriate framework for funding, they also concluded that the NIH does not adequately describe how public health need is assessed, and recommended the use of burdens and costs of diseases in strengthening their assessment and use of health data.[5]

The relationship between burden of disease and research funding is an important metric to consider when determining funding priorities that impact public health. Hospital-acquired infections (HAIs) are associated with a considerable burden of disease. The Centers for Disease Control and Prevention (CDC) estimates that each year in the United States there are about 1.7 million HAIs and 99,000 associated deaths.[6] The mean direct costs of HAIs (in 2005 dollars) range from $1,257 per case for catheter-associated urinary tract infections to $22,875 per case for ventilator-associated pneumonia.[7]

The pathogens that cause the majority of serious HAIs are *Enterococcus faecium, Staphylococcus aureus, Klebsiella pneumoniae, Acinetobacter baumannii, Pseudomonas aeruginosa*, and *Enterobacter *species- commonly referred to as ESKAPE bacteria.[8] Recently though, *Clostridium difficile *has emerged as an important nosocomial pathogen. In 2007, *C. difficile *was ranked as one of the 20 leading causes of death for people aged 65 and older.[9] Thus, this study has included *C. difficile *in the ESKAPE bacteria definition, henceforth known as ESCKAPE bacteria.

The aim of this study was to determine the amount of funding the NIH's National Institute of Allergy and Infectious Diseases (NIAID) allocates to research on antimicrobial nosocomial pathogens, particularly ESCKAPE pathogens and to compare this funding to the burden of disease caused by two ESCKAPE pathogens.

## Methods

### Identifying Grants Using NIH RePORT Database

The NIH Research Portfolio Online Reporting Tools (RePORT) database was used to identify NIH-funded research grants. Searches were done during the month of November 2011 among all available fiscal years for retrospective grant funding data using the search terms "antibiotic resistance," "antimicrobial resistance," and "hospital-associated infection" that included the National Institute of Allergy and Infectious Diseases (NIAID) as the administration or funding agency. Over 11,000 grants were screened to exclude duplicates from the search terms.

### Classifying Grants into Pathogen Categories

Abstracts of the remaining grants were viewed in detail and categorized by the pathogen of interest (e.g. bacteria, virus, fungi, parasites). If the pathogen was classified as bacteria, the grant was further analyzed for drug resistance and type of bacterial species. If unclear, the grant was reviewed by a second reviewer. Bacterial grants were then classified as hospital-associated and non-hospital-associated based on the type of pathogen. Special emphasis was placed on ESCKAPE bacteria.

### Mortality funding

National mortality data for MRSA and *C. difficile *were obtained from prior publications by the CDC's Active Surveillance Core and National Vital Statistics.[9,10] Funding amounts for MRSA and *C. difficile *were then divided by the most recent national mortality statistics to estimate the dollar amount funded per death for each pathogen.

## Results

Regardless of the search term, total amount and number of grants funded by the NIAID have increased during the study period. From FY2007 to FY2008 the total amount increased by approximately 82% and further increased by 22% from FY2008 to FY2009. The number of grants funded from FY2007 to FY2009 increased by 58% and then 16%. Consequently, the average funds per grant steadily increased as well. This trend seems to follow the total NIAID budget (Table 1).

**Table 1:** Number of NIAID Grants and Amounts by Search Term and Year

Search Term	Fiscal Year (FY)	Total Amount ($)	Total Number of Grants	Amount per Grant ($)
**Antibiotic Resistance**	2007	67,860,927	199	341,010
	2008	72,972,153	223	327,229
	2009	101,623,126	282	360,366

**Antimicrobial Resistance**	2007	6,817,017	24	284,042
	2008	136,111,065	241	564,776
	2009	138,905,976	242	573,992

**Hospital-Associated Infection**	2007	105,574,441	183	576,910
	2008	118,117,691	176	671,123
	2009	158,118,571	216	732,030

**Combined**	2007	180,252,385	406	443,971
	2008	327,200,909	640	511,251
	2009	398,647,673	740	538,713

**Approximate NIAID budget**	2007	4,366,000,000		
	2008	4,561,000,000		
	2009	4,569,000,000		

No duplicate grants in each search term

**Figure 1:**
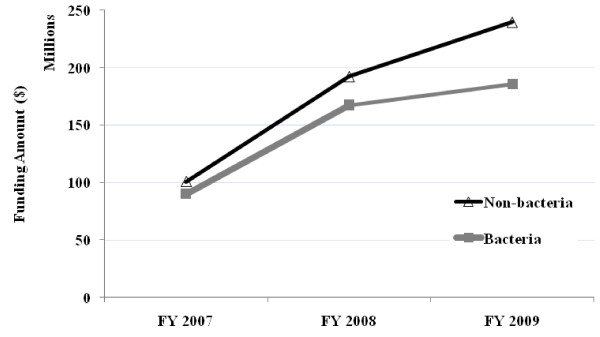
**NIAID Funding for Antimicrobial Resistance Stratified by Non-bacterial Grants vs. Bacterial Grants**.

As mentioned, the funding trend for antimicrobial resistance studies during FY2007 through FY2009 shows a large increase during the first year and then a smaller increase in the subsequent year. This trend is also observed when the grants are stratified by pathogen of interest; non-bacterial (> 90% being viruses) and bacterial (combination of ESCKAPE, Mycobacterium, and other bacterial pathogens) (Figure 1). Both groups show similar overall trend, consistent with the total funds trend, however the funding for bacterial pathogens has seen a smaller increase than non-bacterial pathogens during FY2008 to FY2009.

**Figure 2:**
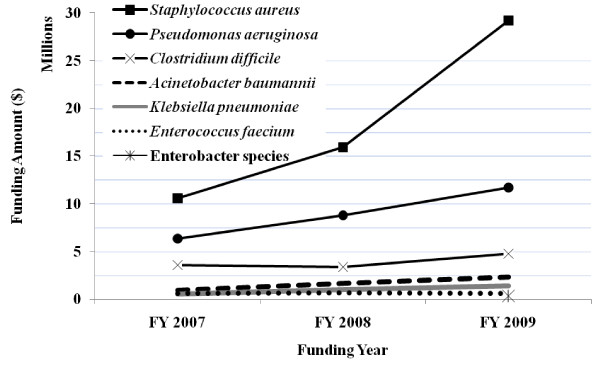
**NIAID Funding by ESCKAPE Pathogen**.

The total funding for all ESKCAPE pathogens was $ 22,005,943 in FY2007, $ 30,810,153 in FY2008 and $ 49,801,227 in FY2009. *Staphylococcus aureus *grants received $ 29,193,264 in FY2009, the highest funding amount of all the ESCKAPE pathogens. Enterobacter species received $ 378,005 in FY2009, the lowest amount of all the ESCKAPE pathogens. In FY2009, *Enterococcus faecium*, *Klebsiella pneumoniae, Acinetobacter baumannii, Pseudomonas aeruginosa*, and *Clostridium difficile *grants received $602,635, $1,412,872, $2,329,872, $11,708,364, and $4,778,850 respectively. The funding trend for all ESCKAPE pathogens increased during the three year study period, except Enterobacter species due to lack of data for years prior to 2009 (Figure 2). Total grant funding increased by 40% for the first year and increased by 61% the following year. The majority of the grant funding was related to *Staphylococcus aureus*, which increased approximately 2.5 times from FY2007 to FY2009.

In 2005, there were an estimated 18,650 deaths among people infected with MRSA.[10] Based on FY2009 funding data for *Staphylococcus aureus*, approximately $1,565 of research funding was spent per death. In 2007, 6,372 people infected with *Clostridium difficile *died.[9] Based on FY2009 funding data, approximately $750 of research funding was spent per *C. difficile *death. According to the NIH RePORT database, NIAID funding for HIV/AIDS was estimated to be $1.302 billion. This is similar to previous reports of $1.244 billion of NIAID funds allocated to HIV/AIDS in 2009.[11] Roughly 17,000 - 18,000 HIV deaths occur annually in the U.S.[12] Based on these rough numbers approximately $72,000 - $76,000 per HIV/AIDS death was spent for research.

**Figure 3:**
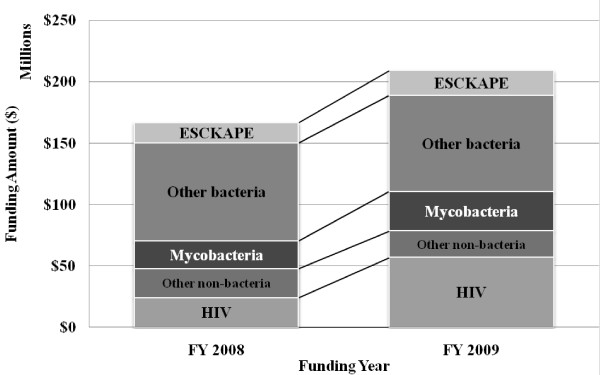
**Funding from NIH Research, Condition, and Disease Categories Category "Antimicrobial Resistance" (FY2008 and FY2009)**.

Our search results were compared to the grants listed in the "Antimicrobial Resistance" category of the NIH RePORT Research, Condition, and Disease Categories (RCDC) (http://report.nih.gov/rcdc/categories/) (Figure 3). Unlike our search, the NIH grants listed in the RCDC Antimicrobial Resistance category only covered the search term "Antimicrobial resistance" for FY2008 and FY2009. However, the overall results are similar; from FY2008 to FY2009 general funding in the RCDC Antimicrobial Resistance category increased by 25%, ESCKAPE pathogen funding increased by 24%, and about half of the funding is devoted to bacterial pathogens. The results that we reported are slightly different from the RCDC results because we included the "Hospital-associated infection" search term, which would have added more non-resistant bacterial grants to our search results.

Using NIH RePORTER we also attempted to evaluate grants funded by other agencies for ESCKAPE pathogens during the FY2007 - 2009. We focused on three major agencies; CDC, the Agency for Healthcare Research and Quality (AHRQ), and Veteran's Health Administration (VHA). No information was obtained for AHRQ and no funding amounts were listed for VHA. During the search, there were only four grants listed for VHA in the FY2009. Nine grants related to ESCKAPE pathogens were funded by the CDC in FY2007-08 and decreased to six grants in FY2009. Funding amounts decreased over the same period from approximately $3 million in FY2007 to approximately $1.9 million in FY2009.

## Discussion

Currently, the antibiotic era is threatened by the convergence of three adverse circumstances: high levels of antibacterial resistance among important pathogens, a dwindling supply of novel classes of antibacterials, and a dramatic reduction in the number of pharmaceutical companies engaged in the discovery and development of antibacterial agents.[13] While 10 new classes of antibiotics were discovered from the 1930s to the 1960s, only two new classes have been approved since 1970.[13] In addition, due to the relatively unfavorable return on investment, pharmaceutical companies have been steering away from engaging in antibiotic-drug discovery.[14,15]

The response to the public-health crisis of antibacterial resistance could be similar to the strategies used to counter HIV. For the past 30 years, NIH-funded research has made great progress in implementing various prevention measures, including reducing the transmission rate of perinatal HIV, HIV drug development and treatment, and a potential vaccine.[16,17] In 2009, the NIH awarded $1.302 billion in research funding for HIV/AIDS.[NIH Research Portfolio Online Reporting Tools (RePORT) http://projectreporter.nih.gov]. As a marker of NIH's HIV research success, in the U.S. in 2005, there were more deaths attributed to MRSA infections than attributable to HIV/AIDS.[18]

The NIH is not blind to the emerging problem of antibiotic resistance, and in response created the 'antimicrobial resistance' funding category. However, the title 'antimicrobial' includes viruses and parasites as well as antibiotic-resistant bacteria. Even though funding continues to increase in the antibiotic-resistance category, we found that only half of the funding is going to antibacterial research and the large increases in funding have targeted non-bacterial pathogens. The ESCKAPE pathogens are inherently different compared to viruses such as HIV and other pathogens such as *Mycobacterium tuberculosis*, thus they require specific attention.[8] From a mortality stand point, even though in the United States MRSA and HIV may be on par with each other, the research dollars invested shows a stark difference ($1,565 vs. $72,000 per death) when only considering NIAID research dollars. Considering other agency dollars, not to mention funding from pharmaceutical companies, will only widen this gap further. In order to have the hope in making the tremendous advances seen in HIV research, antibacterial research would need increased backing. Separate RCDC funding categories in "Antibacterial Resistance" and "Hospital-acquired infections" might be needed in order to highlight the need for more research dollars and attention to this ongoing crisis.

Our estimates may be limited since we only assessed NIAID funding, and not other institutes in the NIH or other agencies such as the CDC, the Agency for Healthcare Research and Quality (AHRQ), or Veteran's Health Administration. However, NIAID is the major agency within NIH that focuses on antimicrobial research. NIAID currently spends $800 million per year in antimicrobial research, which includes $200 million annually in research to better understand the causes, consequences, and treatments of drug resistance.[19] CDC has allocated approximately $17 million for antimicrobial resistance, which is mainly for preparedness, detection, and control purposes not for research.[20] Congress appropriated $5 million for MRSA and related hospital-associated infections in 2007-2008, then in 2009 $17 million ($8 million for MRSA and the remaining for other HAI) to AHRQ.[21] The VHA receives approximately $500 million in medical research but most of the funding is divided among other projects such as Gulf War illnesses, genomics, prosthetics, diabetes, and heart disease.[22] Very little is given to antimicrobial research. The combined total from these various agencies is a fraction of what the NIAID funds, thus our research is representative of the funding situation of antimicrobial research. Our data may be limited by missing information in the NIH RePORT website. However, this is the best public access source of NIAID research funding and should be up to date for FY2007-2009. Finally, our estimates of the amount of research funding spent per *S. aureus *or *C. difficile *death may be an overestimate or underestimate since we do not have mortality data from FY2009. However, the number of deaths associated with MRSA or *C. difficile *should not have significantly declined because no new antibiotics have become available since 2005.

In summary, although it is impressive that the funding for ESCKAPE pathogens has increased over a relatively short period of time, funding levels for antimicrobial-resistant bacteria related grants is still lower than funding for antimicrobial-resistant, non-bacteria related grants. Annual NIAID funding for MRSA and *C. difficile *infections remains below $2,000 per death. Steps should be made in order to match the funding for ESCKAPE pathogens to their disease burden. If we are to have any hope in holding back the tide of antibacterial resistant pathogens as a cause of community and hospital-acquired infections, it is likely that funding will have to increase to the levels successfully employed in HIV-related research during the past three decades. NIAID should be encouraged to make a clear public commitment to increasing funding for the development of new antibacterials and expand the knowledge base around infection prevention.

## Competing interests

The authors declare that they have no competing interests.

## Authors' contributions

SK performed the review, performed the statistical analysis, and wrote the manuscript. MS participated in the study design and coordination. EN conceived of the study, participated in its design and coordination. All authors read and approved the final manuscript.

## References

[B1] Istook E Research funding on major diseases is not proportionate to taxpayers' needs J NIH Res 1997 9 8 26 8

[B2] Anderson C NIH budget: A new kind of earmarking Science 1993 260 5107 483 8475380 10.1126/science.8475380

[B3] Marshall E Lobbyists seek to reslice NIH's pie Science 1997 276 5311 344 6 10.1126/science.276.5311.344 9139352

[B4] National Institutes of Health Working Group on Priority Setting Setting research priorities at the National Institutes of Health 1997 Bethesda, MD: National Institutes of Health

[B5] Committee on NIH Research Priority-Setting Process Scientific opportunities and public needs: Improving priority setting and public input at the national institutes of health 1998 20845560

[B6] Klevens RM Edwards JR Richards CL Jr Horan TC Gaynes RP Pollock DA Cardo DM Estimating health care-associated infections and deaths in U.S. hospitals, 2002 Public Health Rep 2007 122 2 160 6 17357358 10.1177/003335490712200205 PMC1820440

[B7] Perencevich EN Stone PW Wright SB Carmeli Y Fisman DN Cosgrove SE Society for Healthcare Epidemiology of America Raising standards while watching the bottom line: Making a business case for infection control Infect Control Hosp Epidemiol 2007 28 10 1121 33 10.1086/521852 17933084

[B8] Rice LB Federal funding for the study of antimicrobial resistance in nosocomial pathogens: No ESKAPE J Infect Dis 2008 197 8 1079 81 10.1086/533452 18419525

[B9] Xu JQ Kochanek KD Murphy SL Tejada-Vera B Deaths: Final data for 2007. National vital statistics reports 2010 Hyattsville, MD: National Center for Health Statistics 25075874

[B10] Klevens RM Morrison MA Nadle J Petit S Gershman K Ray S Harrison LH Lynfield R Dumyati G Townes JM Craig AS Zell ER Fosheim GE McDougal LK Carey RB Fridkin SK Active Bacterial Core surveillance (ABCs) MRSA Investigators Invasive methicillin-resistant *Staphylococcus aureus *infections in the United States JAMA 2007 298 15 1763 71 10.1001/jama.298.15.1763 17940231

[B11] National Institutes of Health National Institute of Allergy and Infectious Diseases Congresional justification 2008 Bethesda, MD: National Institutes of Health

[B12] Centers for Disease Control and Prevention HIV surveillance report 2008 http://www.cdc.gov/hiv/topics/surveillance/resources/reports/

[B13] Wenzel RP The antibiotic pipeline--challenges, costs, and values N Engl J Med 2004 351 6 523 6 10.1056/NEJMp048093 15295041

[B14] Projan SJ Why is big pharma getting out of antibacterial drug discovery? Curr Opin Microbiol 2003 6 5 427 30 10.1016/j.mib.2003.08.003 14572532

[B15] Carlet J Collignon P Goldmann D Goossens H Gyssens IC Harbarth S Jarlier V Levy SB N'Doye B Pittet D Richtmann R Seto WH van der Meer JW Voss A Society's failure to protect a precious resource: Antibiotics Lancet 2011 378 9788 369 71 10.1016/S0140-6736(11)60401-7 21477855

[B16] Burr CK Lampe MA Corle S Margolin FS Abresh C Clark J National Organizations' Collaborative to Eliminate Perinatal HIV in the US An end to perinatal HIV: Success in the US requires ongoing and innovative efforts that should expand globally J Public Health Policy 2007 28 2 249 60 10.1057/palgrave.jphp.3200126 17585325

[B17] Long EF Owens DK The cost-effectiveness of a modestly effective HIV vaccine in the United States Vaccine 2011 29 36 6113 24 10.1016/j.vaccine.2011.04.013 21510996 PMC3156325

[B18] Bancroft EA Antimicrobial resistance: It's not just for hospitals JAMA 2007 298 15 1803 4 10.1001/jama.298.15.1803 17940239

[B19] Peters NK Dixon DM Holland SM Fauci AS The research agenda of the National Institute of Allergy and Infectious Diseases for antimicrobial resistance J Infect Dis 2008 197 8 1087 93 10.1086/533451 18419527

[B20] FY2009 budget submission center for disease control and prevention discretionary all-purpose table [Internet] Available from: http://www.cdc.gov/fmo/fmofybudget.htm

[B21] Agency for Healthcare Research and Quality AHRQ's efforts to prevent and reduce healthcare-associated infections: FY2009 projects Rockville, MD: Agency for Healthcare Research and Quality

[B22] Panangala SV U.S. Congressional Research Service Veterans medical care: FY2009 appropriations (RL34598; July 29, 2008)

